# Quantitative MRI of Vastus Medialis, Vastus Lateralis and Gluteus Medius Muscle Workload after Squat Exercise: Comparison Between Squatting with Hip Adduction and Hip Abduction

**DOI:** 10.2478/v10078-012-0039-z

**Published:** 2012-07-04

**Authors:** Augusto P. Baffa, Lilian R. Felicio, Marcelo C. Saad, Marcello H. Nogueira-Barbosa, Antonio C. Santos, Débora Bevilaqua-Grossi

**Affiliations:** 1 Departamento de Ciências da Saúde Aplicadas ao Aparelho Locomotor.; 2 Centro de Ciência de Imagens e Física Médica, Faculdade de Medicina de Ribeirão Preto, Universidade de São Paulo, Ribeirão Preto, SP, Brasil.

**Keywords:** quantitative MRI, muscle functional magnetic resonance, proton relaxation time, T2, vastus medialis, gluteus medius, squat exercise

## Abstract

The aim of the present study was to evaluate the use MRI to quantify the workload of gluteus medius (GM), vastus medialis (VM) and vastus lateralis (VL) muscles in different types of squat exercises. Fourteen female volunteers were evaluated, average age of 22 ± 2 years, sedentary, without clinical symptoms, and without history of previous lower limb injuries. Quantitative MRI was used to analyze VM, VL and GM muscles before and after squat exercise, squat associated with isometric hip adduction and squat associated with isometric hip abduction. Multi echo images were acquired to calculate the transversal relaxation times (T2) before and after exercise. Mixed Effects Model statistical analysis was used to compare images before and after the exercise (ΔT2) to normalize the variability between subjects. Imaging post processing was performed in Matlab software. GM muscle was the least active during the squat associated with isometric hip adduction and VM the least active during the squat associated with isometric hip abduction, while VL was the most active during squat associated with isometric hip adduction. Our data suggests that isometric hip adduction during the squat does not increase the workload of VM, but decreases the GM muscle workload. Squat associated with isometric hip abduction does not increase VL workload.

## Introduction

The squat exercise is commonly prescribed in rehabilitation and muscle strength programs by fitness trainers, strength coaches, health professionals and physical therapists (McKean et al., 2010). There is increased interest because this exercise is considered a functional multijoint exercise that simulates more closely daily activities (Boudreau et al., 2009; Besier et al., 2009). There are several types of squat exercises classified in 3 groups: full or deep squat, half or parallel squat and partial or semi squat (Colker et al., 2002; Scaglioni-Solano et al., 2005). The partial squat can be prescribed at the beginning level when the stabilization of the joint and neuromuscular control is the primary goal (Chandler et al., 1989; Stensdotter et al., 2003; Tang et al., 2001). Previous studies of partial squat have focused on vastus medialis and vastus lateralis muscles because they work synergistically to stabilize the patella (Irish et al., 2010; Hodges and Richardson, 1993; Earl et al., 2001; Coqueiro et al., 2005; Hertel et al., 2004). A decreased vastus medialis activity may lead to patella maltracking, increasing the risk of inflammation and premature cartilage degeneration (Irish et al., 2010). However, recently the hip abductors muscles were also considered important for the patella tracking because it is believed that they control the internal femoral rotation during functional activities (Powers, 2003; Dierks et al., 2008). According to Powers (2003), the internal femoral rotation would increase the knee’s dynamical Q angle, increasing the lateral force vector, thus changing the patella tracking. The weakness of the gluteus medius muscle has been observed in other lower extremity injuries also, supporting its stability role not only in the patella, but in the entire lower limb (McHugh et al., 2006; Fredericson et al., 2000; Hiemstra et al., 2005).

Some investigators studying the correction of the patellar tracking with squat exercises argued that, due to the origin of vastus medialis muscle fibers in the magnus adductor, the combination of hip adduction during the quadriceps contraction could increase vastus medialis recruitment (Hodges and Richardson, 1993; Earl et al., 2001). However, Earl et al. (2001) and Coqueiro et al. (2005) did not find significant electromyographic activity differences between vastus medialis and vastus lateralis muscles during squat associated with isometric hip adduction.

Although widely used in kinesiology studies, surface electromyography presents a spatial resolution limitation (Basmajian et al., 1985). However, muscle Quantitative Magnetic Resonance Imaging specifically the Relaxometry can analyze all the muscle cross sectional area and analyze better deeper muscles (Price et al., 1992). There are several investigations using Relaxometry in kinesiological studies, showing its sensitivity to detect changes in the synergic muscle workload pattern (Yue et al., 1994; Ploutz and Dudley, 1992). This quantitative MRI method reflects the detailed changes in the intramuscular water content that increase proportionally with muscle workload (Ono et al., 2011).

Until present, only one paper (Ploutz and Dudley, 1992) has used Relaxometry to analyze muscular workload during squat, even though the authors did not report spin-spin or transversal relaxation time (T2) values for the vastus medialis, vastus lateralis and gluteus medius muscles. There is no previous publication, to our knowledge, analyzing muscle workload by Relaxometry during the squat exercises associated with isometric hip adduction and abduction. Thus the aim of this study was to compare the T2 of the vastus medialis, vastus lateralis and gluteus medius before and after squat exercise associated with isometric hip adduction and abduction. We hypothesized that the addition of isometric hip adduction during squat increases vastus medialis workload, decreasing gluteus medius workload; and the addition of isometric hip abduction during squat increases gluteus medius workload and increases vastus lateralis workload.

## Method

### Design

**Figure 1:**
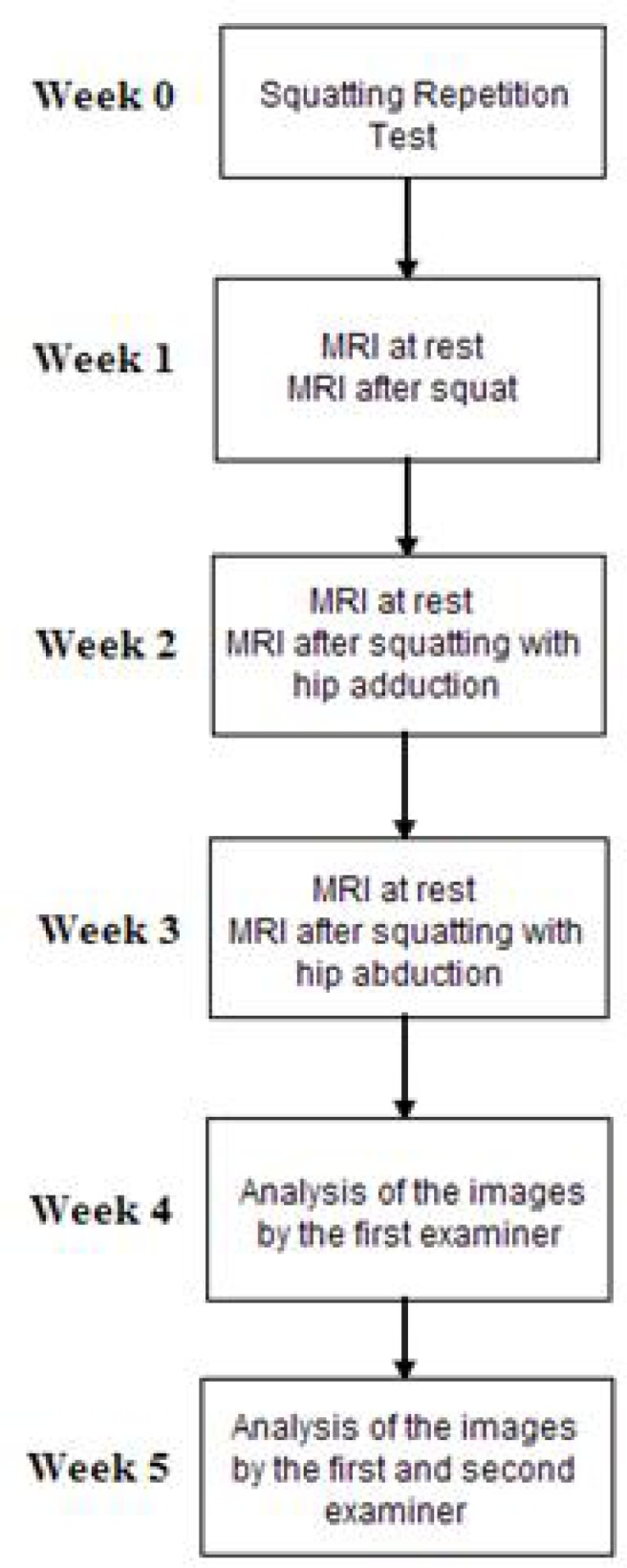
Scheme of the experimental protocol showing the time interval between the events and order of the steps performed by the volunteers and examiners

This experimental, prospective and transversal study used a magnetic resonance scanner Magneton Vision Siemens – 1.5 T (Erlangen, Germany) installed at the same institution of the authors to acquire the images. The average value of the relaxation time (measured in milliseconds) of the region of interest (ROI) in the resting state was compared with the same ROI after exercise to assess the muscle workload. The greater the muscle workload, the greater the difference between the average relaxation time will be (Adams et al., 1992; Conley et al., 1997). As shown in Figure 1, the three exercises were done one week apart between them to minimize fatigue and training effects. After the complete acquisition of the images a blind analysis about the types of squats and periods (resting or post-exercise) was made by a well trained examiner.

### Participants

Fourteen sedentary female volunteers were studied, with no clinical symptoms, average age 22 ±2 years, body height 1.63 ±0.04 m, average body mass 54 ±4 kg, with no previous history of knee lesion or surgery on the knee and lower limbs. Subjects were excluded from the study group if they had reported pain in the anterior knee during physical activity, climbing up and down stairs, squatting, kneeling and prolonged sitting, isometric quadriceps exercises and/or presenting neurological and rheumatological complications. All volunteers were informed about the procedures and signed an informed consent form in accordance with the Ethics Committee for Research Involving Human Beings of the same institution of the authors. There were no dropout from the study and all volunteers had completed all the steps in the same sequence.

### Measures

Proton images using a multi-echo sequence with TR=3,000ms; TE= 22, 60, 120ms; slice thickness 5mm; matrix of 168 × 256, FOV 490 mm and pixel size of 2.55 × 1.91 mm were acquired. Two sets of axial images were taken, first of the hip region and second of the thigh, totaling 44 images, requiring 14 minutes and 20 seconds of data collection. To determine the relaxation times, the images were processed with a software developed in Matlab (Figure 3) (Carneiro et al., 2006). Three axial images located with markers made with vitamin E capsules placed in the same location of the EMG electrodes for the vastus medialis and vastus lateralis muscle according to the norms of Surface Electromyography for the Non-Invasive Assessment of Muscles (SENIAN). The gluteus medius was localized through 22 images above the greater trochanter and only one section with maximum volume was used to analyze it. The ROI was manually selected with a mouse, afterwards the mean between the pixels of the ROI was automatically calculated by the Matlab software. The workload of each muscle was expressed as changes in the relaxation time T2 in milliseconds.

### Procedures

**Figure 2:**
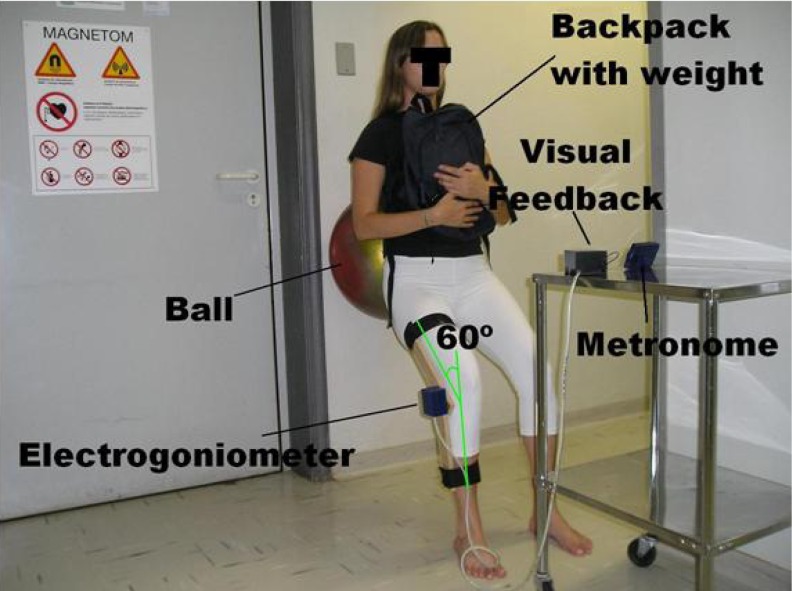
Execution of the squat exercise supported by a ball between the lumbar of the volunteer and the wall of the MRI shielded room. The metronome and light (LED) of the electrogoniometer, which indicates when the maximum flexion was attained, were placed on a table in front of the volunteer

**Figure 3:**
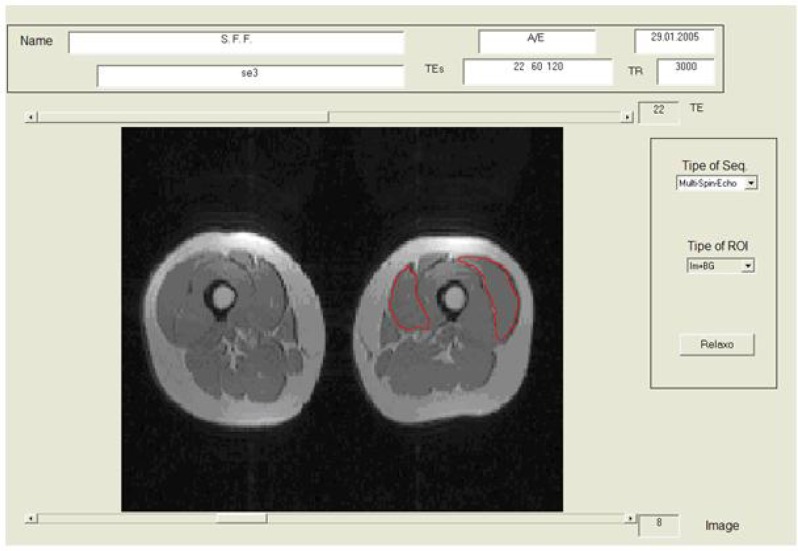
Layout of computer screen of the software Matlab used for the selection of the ROI of vastus medialis and vastus lateralis and to quantify the muscle relaxation time T2

To perform the squat exercise, the volunteer was positioned in a standing position, with her back towards a wall, with the feet separated at a distance equal to the shoulder width. A Gyminic 50-cm-diameter ball (Italy) was held between the volunteer’s lumbar region and the wall to facilitate movements during the exercise and to maintain a vertical alignment of the knees and heels. The trunk was positioned parallel to the wall, avoiding a decrease in the load on the quadriceps muscle or an increase in the lumbar paravertebral musculature, as shown in Figure 2.

The 60º knee flexion was controlled by an electrogoniometer with a visual feedback positioned in front of the volunteer. A backpack loaded with a weight of 25% of the volunteer’s body mass was held over the thorax (Ninos et al., 1997). A metronome produced a cadence of two seconds for each phase of the squat, two seconds up and two seconds down (Korg MA-30, China). The squat was performed as close as possible to the MRI scanner in order to minimize volunteer’s transit time, with data collection starting two minutes after the end of the exercise session (Figure 2) (Nygren and Kaijser, 2002).

The same procedure was used for the squat associated with isometric hip adduction exercises but, in this case, the device to produce adduction resistance was used (Coqueiro et al., 2005). This device was held between the knees articular interline which assured the alignment with the feet and the volunteer was instructed to perform her maximum isometric contraction, in order to constantly compress the device during the flexion and extension of the knee.

To perform the squat associated with isometric hip abduction exercise, a padded Velcro band was attached to the knee articulation line and the volunteer was instructed to perform a maximum isometric contraction forcing the abduction. Every minute, the volunteer was stimulated by verbal command to perform her maximum isometric adduction or abduction during the squat exercise.

A Repetition Maximum Test was applied one week before the images were taken to determine the number of squat exercises to be performed by each volunteer in the three squat series. The simple squat exercise was performed without interruption until the volunteer was unable to continue due to fatigue of the thigh. After ending the test, the volunteer was instructed to complete the CR10 scale of Perceived Effort in the following way: draw a vertical line across a 100mm horizontal line to indicate the global effort, where the extreme left represented no effort and the extreme right a maximum effort (Borg et al., 1998). A series with the half of the maximum squat repetition was adopted for the three types of exercises: squat exercises, squat associated with isometric hip adduction and squat associated with isometric hip abduction. For the Relaxometry analysis the volunteers performed these three exercises in the same order (not randomly) with one week apart between them. The resting images that were compared to the post exercise images were taken in the same day a few minutes before the start of the exercise (Figure 1).

### Analysis

Mixed Effect Model statistical analysis was used to compare the resting images with the ones obtained after the exercise (ΔT2) to normalize the variability between subjects. The mixed effect model is similar to a two way Analysis of Variance (ANOVA) test, but considering the subjects as a random effect (Schall, 1991). Thus the variables for the significance level of p ≤ 0.05 were: type of exercise, muscle, period (resting or post-exercise) and subjects.

## Results

### 

#### Repetition maximum test:

The average number of squat repetitions performed by the volunteers in the Repetition Maximum was 385 ± 170, the average load was 13kg ± 1.43 and the average Borg CR10 scale drawn on the 100 mm line was 81.93 mm ± 9.52.

#### Comparison between rest images with post-exercise images:

All the images post-exercise presented a significant difference to the resting images demonstrating that the load was sufficient to change the muscle metabolism.

#### Comparison between muscle and exercises:

**Figure 4:**
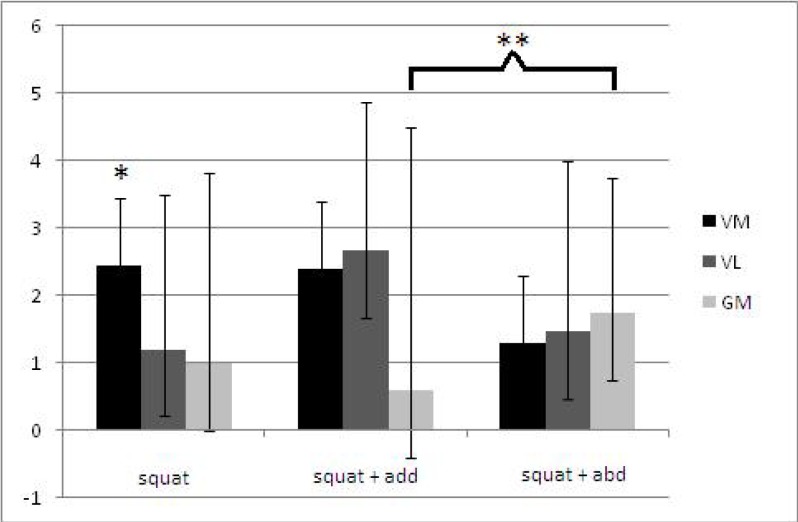
Mean workload and standard deviation in milliseconds (ms), thru the Mixed Effect Model, for each exercise. Vastus Medialis (VM), Vastus Lateralis (VL) and Gluteus Medius (GM). * VM significant different from VL and GM ** GM of squat significant difference from squat associated with isometric hip adduction p ≤ 0.05

For the squatting exercise, the vastus medialis muscle showed significantly higher ΔT2 than the vastus lateralis. Our results show that gluteus medius presented the highest ΔT2 value in the squat associated with isometric hip abduction exercise and significantly different only for the squat associated with isometric hip adduction exercise. Vastus lateralis presented a higher ΔT2 variation for the squat associated with isometric hip adduction exercise, with a significant difference for the other exercises. Unlike the other muscles, vastus medialis showed the smallest ΔT2 in the squat associated with isometric hip abduction exercise (Table 1) (Figure 4).

## Discussion

The use of MRI to quantify muscle function has been extensively investigated in the last three decades, but the exact mechanism for changes in T2 is not known (Saab et al., 2000). In principle, many factors could contribute, including increase in intracellular and extracellular water content, accumulation of diamagnetic ions (eg. lactate, phosphate, sodium) and decrease in pH (Jenner et al., 1994; Meyer and Prior, 2000). Some authors had demonstrated a direct relationship of the increase in T2 with the muscle perfusion and volume, but Prior et al. (2001) had documented the increase of T2 in rat muscle even with the occlusion of the vein and electric stimulation (Ploutz-Snyder et al., 1997). Although Cheng et al. (1995) observed that the recovery of muscle pH after exercise is faster than the recovery of T2.

The Relaxometry method had good applicability, as demonstrated by Yue et al. (1994), only five repetitions of arm curl with a load of 25% of 1 Repetition Maximum were enough to increase the T2 of the short head of biceps brachii, thus proving the sensitivity of the method. In the same way Adams et al. (1992) could find differences in the increase of the T2 of the concentric and eccentric muscle contraction. Previous studies provided evidence of a linear association between the ΔT2 and the exercise intensity suggesting an index of muscle workload (Yue et al., 1994; Meyer and Prior, 2000).

Based in those reports and in our data, it is possible to suggest the type of squat exercise that optimally workload the muscles responsible for stabilizing the patellofemoral joint and the knee dynamic Q angle. The squat exercise presented a significant higher workload of vastus medialis in relation to vastus lateralis, suggesting that the squat exercise presented less risk of patellofemoral maltracking among the 3 squats used in this work. The hypothesis that the contraction of the hip adductor muscles would favor the vastus medialis in the squat associated with isometric hip adduction exercise was not confirmed by our data. There was no workload difference between vastus medialis and vastus lateralis, suggesting that squat associated with isometric hip adduction does not selectively recruit the vastus medialis muscle. But our hypothesis that the gluteus medius workload decrease with the association of isometric hip adduction was confirmed. Squat associated with isometric hip abduction was the only exercise that presented a balance of vastus medialis, vastus lateralis and gluteus medius muscles, despite a gluteus medius workload similar to squat exercise.

A possible explanation for our observation of a non significant difference in gluteus medius workload when compared squat exercise to squat associated with isometric hip abduction exercises could be related to the kind of contraction performed for hip abduction. According to Price et al. (1998), isometric contraction induces a smaller variation of T2 than dynamical muscle contraction. Another factor could be the influence of the hip abduction angle. According to Kumagai et al. (1997), the greater the hip abduction angle, the smaller the T2 alteration of the gluteus medius. The separation of the legs during the squat exercise in the present study was determined by the alignment in the frontal plane of the maleolus lateralis to the acromion, to favor lower limb alignment and not gluteus medius muscle positioning.

Ploutz and Dudley (1992) are the only authors that have analyzed muscular workload by Relaxometry after squat. However, they did not compare vastus medialis and vastus lateralis workload, but only the vastus muscles in a general way with the adductor muscle and the rectus femoralis muscle. Since specific portions of the vastus and the gluteus medius were not analyzed, their results cannot be compared with the ones obtained in the present study. No previous work analyzed gluteus medius workload by Relaxometry after squat exercise.

Due to the lack of previous work analyzing vastus medialis, vastus lateralis and gluteus medius muscle workload by Relaxometry post squat associated with isometric hip adduction, it was only possible to compare the present data with results obtained by surface electromyography. Our results of the comparison between the exercises agree with those found by Cerny (1995), Earl et al. (2001) and Coqueiro et al. (2005), where squat associated with isometric hip adduction did not increase the recruitment of the vastus medialis muscle. However, our data of the comparison between the muscles do not agree. Vastus Medialis showed a stronger workload during squat exercise, while Cerny (1995), Earl et al. (2001) and Coqueiro et al. (2005) reported no significant difference between vastus medialis and vastus lateralis activity for squat exercise. The difference in vastus medialis activity during squat exercise may be due to the difference in the knee flexion angle performed by the volunteers. In the present study, the knee flexion angle was 60º, whereas 45º in Cerny (1995) and Coqueiro et al. (2005), and 30º in Earl et al. (2001). Thus, as has been previously stated in the literature, the increase of the activation of the vastus medialis is directly proportional to the increase of the knee flexion angle during squat (Escamila, 2001).

The temporal resolution is a limitation of the Quantitative MRI because it is not possible yet to measure the muscle activity in real time. Thus the interpretation of the data is related to the whole work performed by the muscle and not to a single activity.

The authors felt that the verbal command minimized the variability between subjects of the degree of voluntary muscle activation of the hip adduction/abduction.

## Conclusion

To summarize, the association of isometric hip adduction during the squat did not increase the workload of vastus medialis, but decreased the gluteus medius muscle workload, suggesting that this type of squat is not beneficial for persons with increased dynamic Q angle. Squat associated with isometric hip abduction did not increased vastus lateralis workload, thus this type of squat could correct the knee dynamic Q angle and avoid patella maltracking. Future strength programs could include squat exercise associated with isometric hip abduction to prevent or correct lower limb imbalance during functional tasks.

## Figures and Tables

**Table 1:** Mean (SD) ΔT2 in milliseconds (ms) during each intervention and mean difference (95% CI) between exercises for each muscle

Outcome	Exercises		Difference between exercises		

	Squat (n = 14)	Squat + adduction (n = 14)	Squat + abduction (n = 14)	Squat + adduction minus Squat	Squat + abduction minus Squat
Gluteus medius	0.98 (2.83)ms	0.59 (3.9)ms	1.75 (2)ms	−0.39 (−3.2 to 0.07)	0.77 (−0.86 to 2.4)
Vastus lateralis	1.2 (2.3)ms	2.67 (2.21)ms	1.47 (2.53)ms	1.47 (0.53 to 2.4)	0.27 (−0.67 to 1.22)
Vastus medialis	2.45 (1.62)ms	2.4 (1.94)ms	1.3 (2.25)ms	−0.05 (−0.99 to 0.89)	−1.15 (−2.09 to −0.2)
